# Mortality Associated with Influenza in Tropics, State of São Paulo, Brazil, from 2002 to 2011: The Pre-Pandemic, Pandemic, and Post-Pandemic Periods

**DOI:** 10.1155/2013/696274

**Published:** 2013-06-12

**Authors:** André Ricardo Ribas Freitas, Priscila M. S. Bergamo Francisco, Maria Rita Donalisio

**Affiliations:** ^1^Epidemiology, Department of Public Health, Faculty of Medical Sciences, State University of Campinas (UNICAMP), 126 Tessália Vieira de Camargo, 13083889 Campinas, SP, Brazil; ^2^São Leopoldo Mandic Medical College, Campinas, SP, Brazil; ^3^Campinas Department of Public Health, Campinas, SP, Brazil

## Abstract

The impact of the seasonal influenza and 2009 AH1N1 pandemic influenza on mortality is not yet completely understood, particularly in tropical and subtropical countries. The trends of influenza related mortality rate in different age groups and different outcomes on a area in tropical and subtropical climate with more than 41 million people (State of São Paulo, Brazil), were studied from 2002 to 2011 were studied. Serfling-type regression analysis was performed using weekly mortality registries and virological data obtained from sentinel surveillance. The prepandemic years presented a well-defined seasonality during winter and a clear relationship between activity of AH3N2 and increase of mortality in all ages, especially in individuals older than 60 years. The mortality due to pneumonia and influenza and respiratory causes associated with 2009 pandemic influenza in the age groups 0–4 years and older than 60 was lower than the previous years. Among people aged 5–19 and 20–59 years the mortality was 2.6 and 4.4 times higher than that in previous periods, respectively. The mortality in all ages was higher than the average of the previous years but was equal mortality in epidemics of AH3N2. The 2009 pandemic influenza mortality showed significant differences compared to other years, especially considering the age groups most affected.

## 1. Introduction

Influenza is a significant cause of mortality in temperate countries [1–3]. There are still many questions concerning the impact of influenza on mortality in the tropics [4–6]. There is also a lot of controversy regarding the quantitative aspects of mortality associated with the 2009 AH1N1 pandemic. It is observed differing severity of disease in many region, as shown by studies conducted in Mexico, France, USA, and other countries using different methodologies [7–11].

The direct measurement of influenza-related mortality is difficult for several reasons [12]. It is a disease with very nonspecific early symptoms; moreover, physicians often do not collect specimens for diagnostic confirmation. In addition, the time period between onset of symptoms and hospitalization is often too large and does not allow a conclusive diagnosis. Another reason is that many patients die from bacterial complications or from decompensation of preexisting conditions, leading to confusion in defining the underlying cause. For these reasons, the basic cause in the death certificate is rarely influenza, although it has been the root cause of the events that led the patient to death [12, 13]. 

 Despite facing these difficulties, the mortality associated with influenza and pneumonia have been analyzed as a marker of viral circulation. Some studies employ statistical models to the time series of others outcomes such as respiratory disease, cardiorespiratory, and all causes of death [12–14]. 

The impact of influenza on mortality is underestimated even during pandemics when there is an effort to increase influenza tests and confirm diagnosis. The study of pneumonia and influenza mortality by age is key to analyze the total burden of disease and to compare with other influenza seasons and with other regions. Understanding the behavior of past pandemics and epidemics of influenza is critical for setting public health priorities for the coming seasonal and pandemic influenza.

The aim of this study is to evaluate the influenza-associated mortality in the State of São Paulo, Brazil, from 2002 to 2011. The choice of this period was due to the availability of systematic virological surveillance data, allowing validate data on mortality associated with influenza with information about the antigenic characteristics and levels of viral activity. 

## 2. Material and Methods

### 2.1. Locality

 São Paulo is the most populate state in Brazil (over 41 million inhabitants in 2010 Census) with a GDP of U.S. $15,000.00 per capita and a Human Development Index of 0.833 (United Nations Development Programme), also with good health care services and epidemiological surveillance [15]. It is located between latitudes 19°46′45′′S and 25°18′43′′S. Despite being in the region of predominantly tropical and subtropical climate, the study region has clearly defined seasons of increased circulation of influenza viruses [16]. 

### 2.2. Mortality and Population

Mortality data were obtained from Health Statistics System (DATASUS), Mortality Information System which covers 100% of the State of São Paulo since 1979 [17]. Causes of death are classified using the International Cause of Death, ICD-10 codes for pneumonia and influenza (ICD J 10 to J18.9), respiratory causes (ICD J00 to J99), and all-cause mortality (excluding external causes of mortality). The mortality rates were calculated in three age groups, 0 to 4, 5 to 19, 20 to 59, and more than 60 years.

Population data were obtained from the Brazilian Institute of Geography and Statistics (IBGE) using data from the 2010 Census [17]. The weekly estimates were obtained by interpolation. 

### 2.3. Virological Data

Data on influenza virus activity in Southeastern Brazil were obtained from the Ministry of Health through the Information System of Epidemiological Surveillance of Influenza Department of Health Surveillance, (SIVEP-Gripe) [18]. This system monitors the occurrence of influenza through sentinel units, which investigate the etiology of respiratory viruses causing flu-like syndromes. National Reference Laboratories test the samples by indirect immunofluorescence for a panel of respiratory viruses (including influenza A and B, parainfluenza 1, 2, and 3, respiratory syncytial virus, and adenovirus) and forward samples for culture of virus and real-time RT-PCR. As the viral subtypes are not provided by SIVEP, data are obtained from various official sources [18–22].

### 2.4. Deaths due to Laboratory-Confirmed Pandemic Influenza

In the beginning of the pandemic, the criterion to confirm the influenza cases was as follows: any patient who had flu-like illness (defined as fever, cough, or sore throat) and history of traveling to countries with occurrence of cases or contacting with infected person. After the initial phase, diffuse transmission was confirmed in epidemiological week 28. At this time the registration of cases was as follows: patients with severe acute respiratory infections (SARI); that is, the definition of SARI included fever, cough and dyspnea, or death. All patients reported by the National System of Surveillance Reportable Disease (SINAN) had respiratory secretion samples collected for performing real-time RT-PCR in the National Reference Laboratories. Data on deaths confirming influenza pandemic were extracted from SINAN by age.

### 2.5. Statistical Analysis

The estimation of influenza-associated mortality was obtained through the classic method Serfling with adaptation to weekly data [23]. To fit regression, we used the total period of 10 years excluding the weeks of greater viral circulation by laboratory criteria.

We defined the onset of periods of increased activity of the influenza virus by virological criteria in the Brazilian southeast (where State of São Paulo is located) when there was the occurrence of two consecutive weeks in which was confirmed by indirect immunofluorescence more than twice of the annual average of cases. We defined that this period ends with the occurrence of two consecutive weeks with viral diagnostic below the annual average. The period of highest viral activity in 2009 began at the time the Brazilian Ministry of Health [15] officially declared epidemiological situation as “widespread viral transmission” until the official end of the pandemic was reported by WHO [24].

A cyclical linear regression was constructed as follow:

(1) Y=β0+β1∗t+β2∗t2+β3∗t3+β4∗sin⁡(2∗π∗t52.17)+β5∗cos⁡⁡(2∗π∗t52.17)+e1,

where *Y* is the mortality rate, *β* is the coefficients of regression, *t* is time in weeks and *t* ^2^ and *t* ^3^ are variables for adjusting the secular trend of the disease.

After adjusting stepwise linear regression, the baseline of expected mortality in the absence of influenza was defined. Using this reference, influenza epidemic periods were demarcated as the periods in which mortality from pneumonia and influenza was above 95% confidence interval predicted by the model for two consecutive weeks; these periods ended when mortality was less than the upper confidence interval for two consecutive weeks. Weekly excess mortality rate was estimated by the difference between the observed and predicted mortality rates by the model during influenza epidemic periods. Season mortality rate was calculated as the sum of weekly excess mortality rate during the year.

The data were analyzed using the statistical program SPSS for Windows, version 13.0, graphics, and data compilation were made using the Microsoft Office Excel 2007. All databases analyzed were not able to do any kind of patient identification to preserve patients' privacy.

## 3. Results

### 3.1. Viral Activity and Excess Mortality due Pneumonia and Influenza

**Figure 1:**
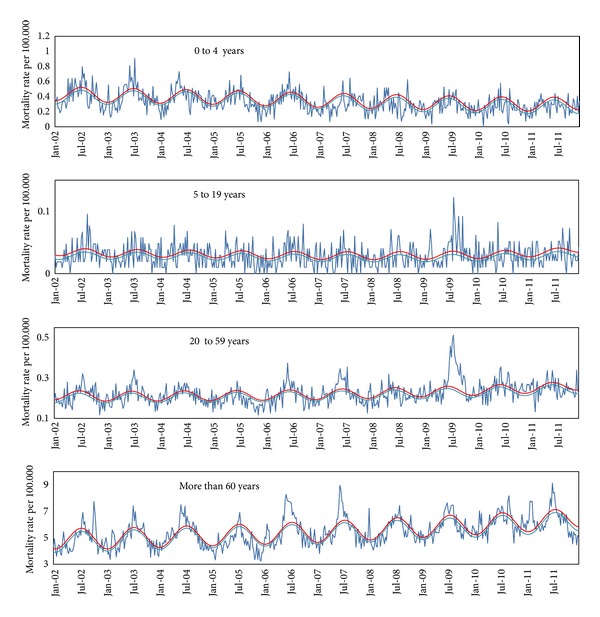
Mortality due to pneumonia and influenza (rate per 100.000). Weekly pneumonia and influenza mortality rate per 100.000 inhabitants by age group, São Paulo, Brazil, January, 2002 to December, 2011. (Dark blue line: observed rate; light blue line: baseline mortality rate predict by model; red line: upper limit of confidence interval).

**Figure 2:**
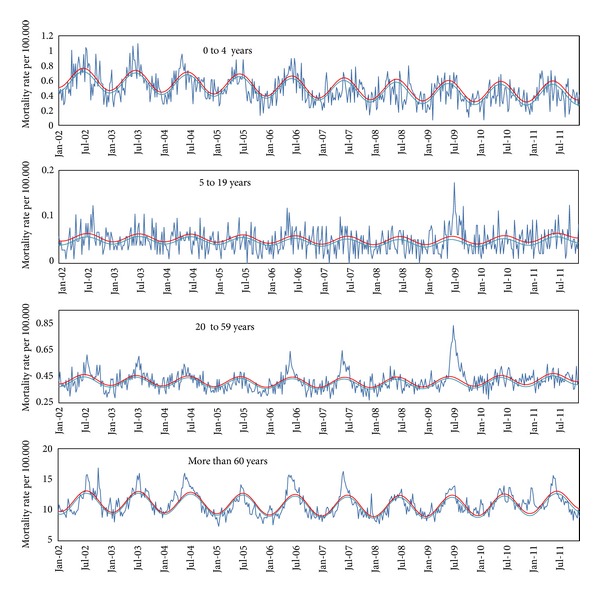
Mortality due respiratory causes (rate per 100.000). Weekly respiratory mortality rate per 100.000 inhabitants by age group, São Paulo, Brazil, January, 2002 to December, 2011. (Dark blue line: observed rate; light blue line: baseline mortality rate predict by model; red line: upper limit of confidence interval).

**Figure 3:**
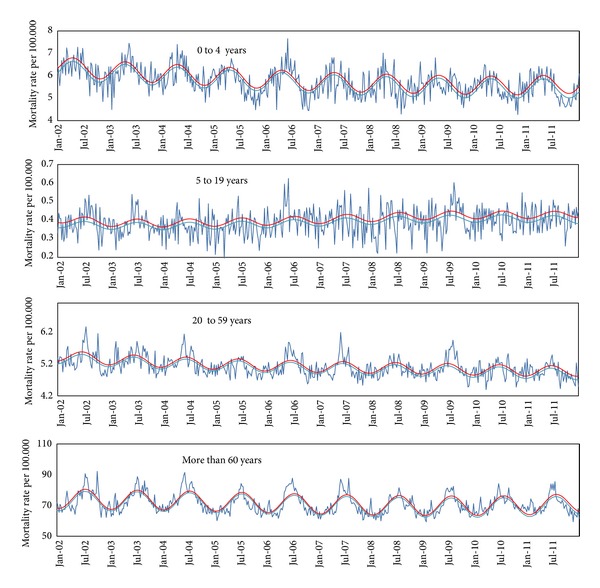
Mortality due to all causes (rate per 100.000). Weekly all causes mortality rate per 100.000 inhabitants by age group, São Paulo, Brazil, January, 2002 to December, 2011. (Dark blue line: observed rate; light blue line: baseline mortality rate predict by model; red line: upper limit of confidence interval).

The weekly mortality due pneumonia and influenza, respiratory causes, and all causes showed a seasonal pattern, with increased mortality during winter in the Southern hemisphere (Figures 1, 2, and 3). There was concurrency between periods of viral activity increased and excess mortality peaks in 8 of the 10 years under study.

In the pre-pandemic period, the years of highest mortality among individuals over 60 years (2006 and 2007) showed high proportion of specimens positive for influenza with predominance of the AH3N2 virus (Tables 1 and 2).

Still considering the pre-pandemic period, the years of lower mortality from pneumonia and influenza in all age groups presented low viral activity (2005) and prevalence of AH1N1 virus (2008), known to be less lethal. 

The first laboratory-confirmed imported cases of influenza AH1N1 pdm 2009 were detected in Brazil in early May. On July 16th, epidemiological week (EW) 28th, Brazilian Health Ministry [15] officially recognized the occurrence of cases due to autochthonous widespread transmission. From early July, EW 26th, there seems to be evidence of excess mortality due to pneumonia and influenza among individuals of 20 to 59 years of age. During weeks EW 28th to 47th in 2009 (late November) we found the vast majority of deaths related to the pandemic first wave, mainly in age groups 5 to 19 and 20 to 59 years.

Along the first half of 2010, there was a predominance of 2009 AH1N1 pdm, while the second AH3N2 variant was more prevalent [19]. In 2010, the excess mortality from pneumonia and influenza in the age group 0 to 4 and 5 to 19 years was, respectively, 0.6 and 0.2 per 100,000, in both cases below the average of previous years. In the age group 20 to 59 years it was 1.1 per 100,000, slightly above the average for seasonal influenza epidemics (0.9 per 100.000), but well below mortality observed in 2009 (2.8 per 100.000). Mortality in over 60 years was 17.0 per 100,000, a level slightly below the average of influenza epidemic years.

That is, in 2010 the overall excess mortality from pneumonia and influenza presented a pattern more like a year of seasonal influenza epidemics, with higher mortality among the elderly and sparing ages between 5 and 59 years, something very different from the pandemic period. This situation may have been influenced by the wide dissemination of the virus in 2009 with naturally induced immunization, extensive vaccination campaign conducted in early 2010 against pandemic influenza and atypical intense circulation of the virus AH3N2 in the second half of 2010. This atypical movement of AH3N2 remained throughout spring of 2010 and early summer of 2011 and may have been a consequence of disturbances in herd immunity caused by the pandemic. 

In early 2011, there was a wave of excess mortality due to pneumonia and influenza in EW 4th and 6th (January and February), probably related to atypical activity of AH3N2. The alternative hypothesis to explain this peak which is the activity of respiratory syncytial virus activity seems unlikely, because the higher prevalence of this virus was in EW 13th that year (SIVEP_GRIPE). The pattern of mortality from pneumonia and influenza in 2011 was similar to years of seasonal H3N2 influenza epidemics with high mortality among the elderly, above normal. 

In 2011, the seasonality seems to have returned to normal, since 86% of positive samples for influenza were obtained in EW 20th to 32th in the Southeast, similar to the standard pattern before the pandemic.

Data from official surveillance for SARI confirmed an excess of mortality like more than half of the estimated cases of deaths in 2009 (54%). The sensitivity for diagnosis appears to have been greater in younger age groups (Table 3). On the other hand, the age group with the highest underreporting was the over 60 years (3%). This may be due to the higher incidence of severe pneumonia in the elderly as a complication of chronic diseases confusing the diagnosis. Moreover, in young patients, viral pneumonia were more severe and clinically distinct from bacterial pneumonia, which is often the cause of complications in patients older than 60 years.

## 4. Discussion

In the study period, we identified pneumonia and influenza excess mortality simultaneously with the increase in viral circulation. There was a clear relationship between the intensity of the circulation of influenza virus known to be pathogenic (AH3N2) and occurrence of mortality from pneumonia and influenza, particularly in over 60 years group. 

The excess mortality due to pneumonia and influenza and other outcomes in 2009 was below the average of previous influenza seasons (2002 to 2008) in the age groups 0 to 4 and over 60 years. In the groups 5 to 19 and 20 to 59 years, during 2009, the pneumonia and influenza excess mortality was, respectively, 2.6 and 4.4 times the average of the previous periods. In all age groups, mortality was higher than those the average of the previous period (2002 to 2008) and equal mortality in epidemics of AH3N2 (2006 to 2007), although the age groups most affected were different (Table 3). The total number of influenza-related deaths in 2009 was higher than the average of previous years, but was lower than in years of seasonal H3N2 influenza epidemics. During epidemics of AH3N2, 86% of deaths from pneumonia and influenza occurred among those over 60 years, while in 2009 pandemic only 36% of the deaths occurred in this age group, confirming the expected shift in age, characteristic of pandemics. These results are consistent with others from study performed in Brazil [25].

Research conducted in other countries shows slightly different results. In The Netherlands increased mortality concentrated in the age group 0 to 4 [26]. The most affected in France were children under 4 and 35 to 44 years (considering the outcome pneumonia, and influenza) [8]. In Austria of all age groups below 44 years had higher mortality than the average of previous years, but the most affected group was children under 14 years [27]. In Mexico, the age groups most affected were 5 to 19 and 20 to 59 years, with increases of 9 and 14.5 times from the average of the previous periods, respectively. The same groups were the most affected in Brazil, although with higher incidence and mortality rates in Mexico [7]. In that country, children under 5 years and elderly older than 60 years were less affected than in previous years, but the influenza pneumonia excess mortality in all ages was 2.6 times higher than that observed in previous years. Study carried out in Hong Kong [9] showed a different tendency, as the most affected by influenza A H1N1 was the elderly. These results should be viewed with caution because it is a unique city. 

Unlike what happened in England, where the second wave seems to have been more intense than the first, in Brazil there was not a second wave of the 2009 pandemic [11].

In Mexico, Charu and colleagues [7] observed increased mortality among people over 60 already in the year 2010, as noted in São Paulo in 2011. In that country, this phenomenon occurred in a period slightly different from the normal influenza seasonal, probably due to an increase in the circulation of the virus AH3N2 which could not be identified by sentinel surveillance due to be concentrated in some region or in certain age groups [7]. 

This study has some limitations mentioned below. As it is an ecological study of mortality rates, certainly there are variables not controlled, as vaccination, climatic changing, and circulation of others virus. Other limitation is the small number of specimens collected weekly (average of 50.0 per week) which may have hampered the identification of small peaks of viral activity, contributing to the lack of perfect synchronization between the excess mortality and increased viral activity. Analysis of subtypes circulating in influenza seasons was compromised by having used aggregate data obtained from the entire South America 2002, 2003, 2005, 2010, and 2011 and may not accurately reflect the local reality of the state of São Paulo (PAHO, WHO). 

This study concludes that the method Serfling adapted to weekly information, with validation through viral activity data using the influenza and pneumonia excess mortality, may be appropriate in this geographic, climatic, and epidemiological context. In the state of São Paulo, mortality from 2009 pandemic influenza affected most age groups 5–50 years, and spared those younger than 5 and older than 60 years. The 2009 influenza H1N1 pandemic had almost all its effect in 2009, without a second significant wave. Others studies are needed with standardized methodology for evaluating the appropriate charge of the 2009 pandemic in different regions considering climatic and social context, health systems, and measures taken. This can be useful to health authorities in developing appropriate contingency plans for new pandemics.

## Figures and Tables

**Table 1:** Annual excess mortality rate per 100.000 inhabitants by age group in prepandemic, pandemic, and postpandemic periods, State of São Paulo, Brazil, 2002 to 2011.

		1–4 years (95% C.I.)	5–19 years (95% C.I.)	20–59 years (95% C.I.)	60 and more years (95% C.I.)	All ages (95% C.I.)
2002	Pneumonia and influenza	2,0 (1,5–2,5)	0,3 (1,2–0,4)	0,7 (0,6–0,9)	16,8 (12,6–21,0)	2,2 (1,9–2,7)
	Respiratory causes	2,7 (2,0–3,4)	0,4 (0,3–0,5)	1,3 (9,9–1,6)	27,0 (21,0–33,0)	3,5 (7,6–4,3)
	All causes	5,5 (3,8–7,2)	1,2 (0,8–1,6)	6,3 (4,8–7,7)	89,1 (61,1–117,0)	12,2 (8,7–15,7)

2003	Pneumonia and influenza	2,5 (2,0–3,0)	0,2 (1,3–0,2)	0,9 (0,7–1,1)	14,6 (11,3–17,9)	2,1 (1,9–2,5)
	Respiratory causes	2,3 (1,9–2,7)	0,3 (0,2–0,3)	1,3 (10,0–1,7)	26,1 (20,7–31,5)	3,3 (7,5–4,0)
	All causes	8,3 (6,0–10,5)	0,9 (0,7–1,1)	5,1 (4,1–6,2)	80,5 (60,2–100,8)	11,0 (8,3–13,6)

2004	Pneumonia and influenza	2,3 (1,8–2,9)	0,2 (1,2–0,3)	0,6 (0,5–0,8)	20,6 (15,6–25,6)	2,4 (2,1–3,0)
	Respiratory causes	2,4 (1,9–3,0)	0,2 (0,2–0,3)	1,2 (10,1–1,7)	47,7 (38,3–57,1)	5,2 (9,2–6,4)
	All causes	9,1 (7,1–11,1)	0,7 (0,6–0,9)	5,5 (4,4–6,6)	98,3 (65,8–130,9)	12,8 (9,0–16,5)

2005	Pneumonia and influenza	0,7 (0,5–1,0)	0,2 (1,2–0,2)	0,1 (0,1–0,2)	2,3 (1,6–3,0)	0,4 (0,6–0,5)
	Respiratory causes	0,4 (0,2–0,6)	0,2 (0,1–0,2)	0,1 (10,3–0,1)	4,8 (3,6–5,9)	0,6 (6,0–0,7)
	All causes	2,8 (1,8–3,9)	0,4 (0,3–0,4)	1,2 (0,7–1,8)	19,8 (15,6–24,1)	2,8 (2,0–3,6)

2006	Pneumonia and influenza	1,6 (1,2–2,0)	0,3 (1,1–0,4)	0,8 (0,6–1,0)	26,3 (22,4–30,2)	3,0 (2,8–3,5)
	Respiratory causes	2,5 (1,8–3,1)	0,4 (0,4–0,5)	1,3 (10,5–1,5)	35,8 (30,8–40,7)	4,2 (8,8–4,9)
	All causes	9,7 (7,4–12,0)	1,8 (1,4–2,2)	5,8 (4,8–6,9)	110,9 (89,2–132,6)	14,4 (11,6–17,2)

2007	Pneumonia and influenza	1,5 (1,1–1,9)	0,1 (1,1–0,2)	1,0 (0,8–1,2)	18,2 (15,0–21,4)	2,6 (2,3–3,0)
	Respiratory causes	2,1 (1,6–2,6)	0,3 (0,2–0,3)	1,6 (10,8–1,9)	25,6 (21,5–29,6)	3,8 (8,6–4,5)
	All causes	5,8 (4,9–6,8)	1,4 (1,1–1,7)	5,1 (4,2–6,0)	87,9 (68,4–107,5)	12,8 (10,1–15,4)

2008	Pneumonia and influenza	1,7 (1,3–2,1)	0,2 (1,1–0,3)	0,4 (0,3–0,5)	7,1 (5,3–9,0)	1,2 (1,1–1,5)
	Respiratory causes	2,1 (1,5–2,8)	0,2 (0,1–0,3)	0,5 (11,2–0,7)	17,0 (12,4–21,6)	2,3 (7,9–3,0)
	All causes	9,2 (6,9–11,6)	0,8 (0,6–1,1)	3,3 (2,3–4,2)	38,8 (26,8–50,9)	6,9 (4,8–9,0)

2009	Pneumonia and influenza	0,9 (0,5–1,2)	0,6 (1,1–0,7)	2,8 (2,4–3,1)	13,1 (9,6–16,6)	3,3 (2,8–3,9)
	Respiratory causes	2,0 (1,3–2,6)	0,8 (0,7–1,0)	3,9 (11,6–4,3)	22,2 (15,4–28,9)	5,0 (8,7–6,1)
	All causes	9,8 (6,7–12,9)	1,3 (0,9–1,7)	8,3 (6,5–10,0)	62,7 (47,8–77,5)	12,7 (9,7–15,6)

2010	Pneumonia and influenza	0,6 (0,4–0,8)	0,2 (1,1–0,3)	1,1 (0,8–1,4)	17,0 (12,5–21,5)	2,7 (2,2–3,4)
	Respiratory causes	1,4 (0,9–1,9)	0,4 (0,3–0,5)	1,4 (12,0–2,0)	34,5 (26,2–42,8)	5,0 (10,2–6,3)
	All causes	5,0 (3,5–6,5)	0,7 (0,5–0,9)	5,7 (4,1–7,2)	106,8 (76,0–137,6)	16,2 (11,5–20,8)

2011	Pneumonia and influenza	1,0 (0,6–1,3)	0,2 (1,2–0,3)	0,8 (0,6–1,0)	20,1 (16,9–23,3)	2,9 (2,6–3,5)
	Respiratory causes	2,3 (1,6–3,0)	0,2 (0,1–0,2)	1,2 (12,4–1,5)	37,6 (30,9–44,3)	5,2 (11,0–6,3)
	All causes	5,5 (3,6–7,4)	1,2 (0,7–1,7)	4,1 (2,9–5,2)	96,2 (76,6–115,9)	14,1 (11,0–17,3)

Average of epidemics H3N2 (2006-2007) years (a)	Pneumonia and influenza	1,5	0,2	0,9	22,2	2,8
	Respiratory causes	2,3	0,4	1,5	30,7	4,0
	All causes	7,8	1,6	5,5	99,4	13,6

Average 2002 to 2008 years (b)	Pneumonia and influenza	1,8	0,2	0,6	15,1	2,0
	Respiratory causes	2,1	0,3	1,1	26,3	3,3
	All causes	7,2	1,0	4,6	75,1	10,4

Rate ratio (2009/a)	Pneumonia and influenza	0,6	2,6	3,2	0,6	1,2
	Respiratory causes	0,9	2,3	2,6	0,7	1,2
	All causes	1,3	0,8	1,5	0,6	0,9

Rate ratio (2009/b)	Pneumonia and influenza	0,5	2,6	4,4	0,9	1,7
	Respiratory causes	0,9	2,9	3,7	0,8	1,5
	All causes	1,4	1,2	1,8	0,8	1,2

**Table 2:** Influenza virus identified by year, positivity of specimens by season State of São Paulo, 2002–2011.

Year	Virus probably predominant	Positive specimens, average in season^6^	Total number of specimens
2002^1^	B (58%), AH3N2 e AH1N1 (20% each)	9.9%	892
2003^1^	AH3N2 (60,6%), H1N1 (27%)	11.8%	1365
2004^2^	AH3N2 (67%), influenza B (20%)	9.0%	2159
2005^1^	H3N2 (65,6%), B (24%) e H1N1 (11,4%)	4.9%	1612
2006^3^	AH3N2	10.4%	2135
2007^3^	AH3N2	8.5%	4840
2008^4^	AH1N1 e B	6.6%	6303
2009^4^	AH1N1 e AH1N1 pdm 2009	7.8%	1703*
2010^5^	AH1N1 pdm2009 = 1st mid, AH3N2 = 2nd mid	4.8%	2205*
2011^5^	AH3N2 e AH1N1 pdm2009	3.5%	2795

^1^FluNet (WHO, data referring to South America) [22].

^2^Guia de vigilância epidemiológica. Ministério da Saúde, Secretaria de Vigilância em Saúde. 6. ed. Brasília 2005 [28].

^3^Boletim da Saúde, 2009 (State Board of Health, Rio Grande do Sul) [29].

^4^Boletim Epidemiológico, 2011 (State Board of Health, Rio Grande do Sul) [30].

^5^Site: http://ais.paho.org/phip/viz/ed_flu.asp [19].

^6^SIVEP_GRIPE-(Brazilian Ministry of Health) [31].

*During the pandemic there was a commitment in the collection of samples for surveillance of flu-like syndromes.

**Table 3:** Deaths by laboratory–confirmed 2009 pandemics and estimates from statics models State of São Paulo, 2002–2011.

	Laboratory-confirmed		Laboratory-confirmed/estimate deaths due to respiratory causes (%)	2009 Pandemics excess mortality rate/100.000 (95% C.I.)		H3N2 epidemics excess mortality rate/100.000 (2006-2007), (95% C.I.)	
	Mortality (rate/100.000)	Deaths		Deaths (P & I)	Respiratory causes	Deaths (P & I)	Respiratory causes
0–4 years	1.4	40	73%	25 (15–35)	55 (36–75)	44 (33–55)	65 (48–82)
5–19 years	0.6	57	70%	58 (49–67)	81 (66–96)	22 (18–27)	35 (28–42)
20–59 years	1.8	418	46%	659 (577–741)	907 (792–1,023)	207 (165–249)	343 (280–407)
60+	0.7	33	3%	425 (318–532)	976 (678–1,274)	962 (814–1,111)	1,351 (1,151–1,550)
All ages	1.4	1098	54%	1,172 (962–1,382)	2,032 (1,581–2,483)	1,117 (927–1,307)	1,627 (1,362–1,892)
Proportion of excess death among >60 years		6%		36%	48%	86%	83%
